# Evidence that Prefibrotic Myelofibrosis Is Aligned along a Clinical and Biological Continuum Featuring Primary Myelofibrosis

**DOI:** 10.1371/journal.pone.0035631

**Published:** 2012-04-20

**Authors:** Giovanni Barosi, Vittorio Rosti, Elisa Bonetti, Rita Campanelli, Adriana Carolei, Paolo Catarsi, Antonina M. Isgrò, Letizia Lupo, Margherita Massa, Valentina Poletto, Gianluca Viarengo, Laura Villani, Umberto Magrini

**Affiliations:** 1 Laboratory of Clinical Epidemiology and Centre for the Study of Myelofibrosis, IRCCS Policlinico S. Matteo Foundation, Pavia, Italy; 2 Biotechnology Research Area, IRCCS Policlinico S. Matteo Foundation, Pavia, Italy; 3 Unit of Clinical Immunology, Immunohematology, and Transfusion Service, IRCCS Policlinico S. Matteo Foundation, Pavia, Italy; West Virginia University School of Medicine, United States of America

## Abstract

**Purpose:**

In the WHO diagnostic classification, prefibrotic myelofibrosis (pre-MF) is included in the category of primary myelofibrosis (PMF). However, strong evidence for this position is lacking.

**Patients and Methods:**

We investigated whether pre-MF may be aligned along a clinical and biological continuum in 683 consecutive patients who received a WHO diagnosis of PMF.

**Results:**

As compared with PMF-fibrotic type, pre-MF (132 cases) showed female dominance, younger age, higher hemoglobin, higher platelet count, lower white blood cell count, smaller spleen index and higher incidence of splanchnic vein thrombosis. Female to male ratio and hemoglobin steadily decreased, while age increased from pre-MF to PMF- fibrotic type with early and to advanced bone marrow (BM) fibrosis. Likely, circulating CD34+ cells, LDH levels, and frequency of chromosomal abnormalities increased, while CXCR4 expression on CD34+ cells and serum cholesterol decreased along the continuum of BM fibrosis. Median survival of the entire cohort of PMF cases was 21 years. Ninety-eight, eighty-one and fifty-six percent of patients with pre-MF, PMF-fibrotic type with early and with advanced BM fibrosis, respectively, were alive at 10 years from diagnosis.

**Conclusion:**

Pre-MF is a presentation mode of PMF with a very indolent phenotype. The major consequences of this contention is a new clinical vision of PMF, and the need to improve prognosis prediction of the disease.

## Introduction

Primary myelofibrosis (PMF) is recognized as a distinct clinical entity among classical Philadelphia-negative myeloproliferative neoplasms (MPNs) which also include essential thrombocythemia (ET) and polycythemia vera (PV) [1]. In spite of a wide range of functional [2] and clinical presentations [3], its uniqueness has been traditionally grounded on the constitutive association of reticulin or collagen fibrosis in bone marrow (BM) with megakaryocyte hyperplasia and dysplasia, and mobilization of hematopoietic progenitor cells with extramedullary hematopoiesis [4]–[6]. In the 90s', Thiele and co-workers disrupted the dogma of BM fibrosis being an intrinsic and necessary stigma of PMF, and they first proposed a new category of patients characterized by absence of relevant reticulin fibrosis in BM with dual megakaryocytic and granulocytic myeloproliferation associated with characteristic megakaryocyte dysplasia [7]–[9]. This variant, called prefibrotic myelofibrosis (pre-MF), has been included as a prodromic phase of PMF into the WHO classification of MPNs since 2001 [10], and the criteria for the diagnosis were further outlined in 2008 [11]. Now, pre-MF has joined the ranks of the diagnostic categories used in the practice of most of the hematopathologists worldwide, even though there is still a number of unresolved issues concerning its diagnostic reproducibility [12]–[16], and molecular and biological identity [17]–[20].

Since the phenotype of pre-MF resembles that of ET, in the last years the research on the disease has established the stronger tendency of patients with pre-MF to have bleeding [21], to evolve into overt PMF and leukemia [22], [23], and to have shorter survival with respect to ET [22], [23].

In this paper we aimed to question whether there is an underlying structure of the patients' clinical and biological data that allows to align pre-MF patients along a continuum of characteristics featuring PMF or to assign it to a separate disease entity among MPNs. We reasoned that the evidence for pre-MF being a prodromic phase of PMF is limited to the histological documentation of a tendency to myelofibrotic progression [7]–[9], [22], [23]. Moreover, the definition of pre-MF in the most recent studies included both pre-MF with zero grade BM fibrosis and early myelofibrosis [22], [23], i.e. with grade 1 BM fibrosis, thus mixing two possibly different categories of patients with different phenotypes and evolutions. Finally, a great deal of evidence on the natural history of pre-MF derives from series collected from patients with an initial clinical diagnosis of ET, and this may limit the spectrum of presentations of pre-MF [22], [23]. To this aim, we will describe the epidemiological, clinical and biological features of the consecutive cases of pre-MF collected among a large cohort of patients diagnosed with PMF in our centre after revision of the diagnostic BM biopsy.

## Methods

### Ethics Statement

The study was approved by the IRCCS Policlinico S. Matteo Foundation's institutional review board. Written informed consent was obtained from each patient before data were entered in the database.

### Study Cohort

The cohort of patients from whom the cases of pre-MF were extracted was composed by all consecutive patients seen from 1990 to 2011 in our centre who received a diagnosis of PMF according to the WHO criteria [10], [11]. The cohort was established after a systematic revision of BM biopsies taken at diagnosis of near all the MPNs cases observed in our centre.

### Data collection

We have collected uniform information about all patients who have been examined at least once as outpatients or inpatients in our centre. Information was collected in a retrospective fashion at the time of first visit, and prospectively thereafter. Patients at diagnosis had complete blood count and differentials, serum cholesterol and lactic dehydrogenase (LDH), physical examination and clinical complications at or preceding diagnosis. Patients evaluated after the diagnosis had these details reviewed from other centres. All patients had BM biopsy at diagnosis or had BM samples taken at diagnosis reviewed by our pathologist (UM). On this sample, the degree of BM cellularity in paraffin-embedded BM biopsy specimens stained with hematoxylin and eosin was estimated. In addition, Gomori stains were applied to paraffin sections of the BM biopsy specimens and quantitative estimation of degree of fibrosis was performed according to the EUMNET criteria [24]. After year 1999, all patients received the enumeration of CD34-positive cells in peripheral blood [6], and the measurement of CXCR4 expression on circulating CD34-positive cells [25]. After year 2005, patients received the assessment of *JAK2*V617F mutation in their peripheral blood granulocytes [26]. In the great majority of the cases the quantitative allele burden was also measured [26]. Chromosomal analysis was not systematically planned, but it was done in few centers and in all patients who received a BM biopsy in our centre. Abnormal karyotype was defined by the presence of at least two metaphases with structural abnormalities or monosomy or three metaphases with polysomy.

### Diagnosis of prefibrotic myelofibrosis

For classifying a patient as having pre-MF we reinterpreted the criteria reported in 2001 by the WHO system [10], and we based the diagnosis on the existence of three bone marrow characteristics: dual myeloid megakaryocyte dominance, megakaryocyte morphology (anisocytosis, dense nuclei with plump lobation) with clustering, and a BM fibrosis grade 0 or less than 1. Patients with the bone marrow morphological features of pre-MF but having at least grade 1 fibrosis were categorized into the category of PMF, fibrotic type.

### Statistical Analysis

Categorical variables are presented as frequencies and percentages, and continuous variables as means and standard deviations, or medians for variables with skewed distribution. Univariate analyses comparing subject characteristics were done with the t test for continuous variables, or by non parametric analysis of variance (Kurskall-Wallis test) or by Mann-Witney U test. The continuum hypothesis that the patient's characteristics varied along three grades of BM fibrosis was tested by verifying if the means of the ordered groups change in a linear or higher order fashion (ANOVA with trend).

Logistic regression was used to assess the ability of the patients' characteristics to predict hematological and biological relevant parameters. We examined the following covariates: sex, age, presence of splanchnic vein thrombosis, *JAK2*V617F mutation. Logistic models were obtained by performing a backward elimination with a P value cut-off of .05, and then allowing any variable previously deleted to enter the final model if its P value was <.05. Survival analysis was drawn using the Kaplan-Meier procedure. Results were considered statistically significant when P values were less than 0.05. All computations were performed with STATISTICA© software (Statsoft, Tulsa, OK, USA).

## Results

### Clinical Characteristics of Prefibrotic Myelofibrosis

Among the 683 patients with PMF, 132 fulfilled the criteria we established for the diagnosis of pre-MF, thus giving an overall frequency of 19.3%. The clinical characteristics of PMF patients at diagnosis stratified according to the category of pre-MF and PMF-fibrotic type are illustrated in Table 1. As compared with patients with PMF-fibrotic type, patients with pre-MF had significantly younger age and higher female to male ratio (P<0.001 for each comparison). Diagnosis of pre-MF occurred at the age of 50 years or younger in 87%, while in 35.6% (P<0.001) in PMF-fibrotic type. As compared with patients with PMF-fibrotic type, patients with pre-MF had higher hemoglobin concentrations (mean increase, 2.3 g/dL, 95% CI, 1.7–2.8 g/dL; P<0.001), higher platelet count (mean increase, 218×10^9^/L, 95% CI, 153–282×10^9^/L; P<0.001), lower white blood cell count (mean decrease, 1.4×10^9^/L; 95% CI, 0.04–2.8×10^9^/L; P<0.052), and smaller spleen index (mean decrease, 63 cm^2^; 95% CI, 41–83 cm^2^; P<0.001). Patients with pre-MF had also lower percentage of circulating immature myeloid cells and no patient had circulating blasts at diagnosis. By using the International Working Group prognostic classification [27], patients with pre-MF displayed a greater frequency of zero-grade score than patients with PMF-fibrotic type (91.3% vs. 51.7%; P = 0.001). Pre-MF patients showed a higher frequency of thrombotic events at the time of diagnosis or in the year preceding the diagnosis than patients with PMF-fibrotic type (30.3% vs. 6.7%; P<0.001) (Table 2). Both in pre-MF and in PMF-fibrotic type, the great majority of the thrombotic events was in splanchnic veins.

**Table 1:** Clinical and laboratory features at diagnosis of 132 patients with prefibrotic myelofibrosis compared with those with primary myelofibrosis-fibrotic type.

	Myelofibrosis-fibrotic type	Prefibrotic myelofibrosis	P§	Myelofibrosis-fibrotic type with BM fibrosis grade 1	P§§	Myelofibrosis-fibrotic type with BM fibrosis grade 2 or 3	P§§§
Number	551	132		206		345	
Percentage (95% CI)	80.7 (77.6–83.7)	19.3 (16.3–22.4)		30.2 (26.8–33.8)		50.5 (46.8–54.4)	
**Demographics**							
Female, number (%)	184 (33.4)	79 (60.3)	0.001	87 (42.2)	0.021	97 (28.1)	0.046
Age at onset of the disease, years, mean (range)	54.9 (6–90)	38.3 (6–73)	<0.001	50.9 (6–80)	<0.001	57.4 (11–90)	<0.001
**Laboratory and Clinical features at diagnosis**							
Hemoglobin, g/L; mean (range)	11.9 (3–19.6)	14.2 (10–20.4)	<0.001	13.3 (4.6–19.6)	0.001	11.1 (3–19)	<0.001
WBC count, ×10^9^/L; mean (range)*	10.6 (1.5–64.3)	9.2 (2.5–31.5)	0.043	11.5 (2.2–50.9)	0.001	10.1 (1.5–64.3)	0.043
Plt count, ×10^9^/L; mean, (range)	432 (5–2926)	647 (115–2700)	<0.001	553 (19–1860)	0.013	359 (5–2926)	<0.001
Spleen index, cm^2^; mean (range)**	181 (70–1200)	118 (80–315)	<0.001	152 (70–627)	<0.001	199 (80–1200)	<0.001

§ P value refers to the difference between prefibrotic myelofibrosis and myelofibrosis-fibrotic type;

§§ P value refers to the difference between prefibrotic myelofibrosis and myelofibrosis-fibrotic type with bone marrow (BM) fibrosis grade 1;

§§§ P value refers to the difference between myelofibrosis-fibrotic type with BM fibrosis grade 1 and with BM fibrosis grade 2 or 3;

* White-blood cell (WBC) count was corrected for the number of circulating erythroblasts;

** Spleen index is the product of the longitudinal by the transverse spleen axis, the latter defined as the maximal width of the organ.

**Table 2:** Major thrombotic events at diagnosis or in the year before diagnosis.

	Prefibrotic myelofibrosis (N = 132)	Myelofibrosis fibrotic type (N = 551)	P
Patients with thrombosis at diagnosis or in the year before diagnosis, N (%)	40 (30.3)	37 (6.7)	0.021
Type of thrombosis			
Splanchnic vein thrombosis, N (%)	31 (77.5)	29 (78.4)	
Budd-Chiari syndrome, N (%)	3 (7.5)	1 (2.7)	
DVT-TE, N (%)	2 (5)	4 (10.8)	
Arterial thrombosis, N (%)	2 (5)	3 (8.1)	
Cerebral sinus thrombosis, N (%)	2 (5)	0 (0)	

### Biological Characteristics of Prefibrotic Myelofibrosis

As compared with patients with PMF-fibrotic type, those with pre-MF had a statistically significant difference in the value of biological parameters we measured as markers of disease activity (Table 3). The greatest differences were obtained for CD34-positive cells frequency and serum LDH value. By contrast, neither the proportion of *JAK2*V617F mutated patients nor the V617F allele burden was significantly different in patients with pre-MF or PMF-fibrotic type. Cytogenetic findings at diagnosis were abnormal in 13.8% of patients with pre-MF while they were abnormal in 35.4% of patients with PMF-fibrotic type (P = 0.034).

**Table 3:** Biological parameters and *JAK2*V617F status.

	All Patients	Primary myelofibrosis- fibrotic type	Prefibrotic myelofibrosis	P§	Primary myelofibrosis-fibrotic type with BM fibrosis grade 1	P§§	Primary myelofibrosis-fibrotic type with BM fibrosis grade 2 or 3	P §§§
**Biological parameters**								
Serum LDH, U/L; median (range)	N = 464, 661 (120–3444)	N = 380, 743 (120–3444)	N = 84, 451 (149–1145)	<0.001	N = 137, 597 (156–2645)	<0.001	N = 243, 826 (120–3444)	<0.001
CD34+ cell in PB, ×10^6^/L; median (range)	N = 276, 13.8 (0.78–1902)	N = 213, 24.6 (0.81–1902)	N = 63, 3.24 (0.78–27)	<0.001	N = 82, 17.5 (0.81–800)	<0.001	N = 131, 34.3 (0.88–1902)	<0.002
CXCR4 on CD34+ cell, %; median (range)	N = 98, 29.4 (1.7–93.2)	N = 79, 25.0 (1.7–93.2)	N = 19, 46.5 (7.1–84.6)	0.035	N = 23, 46.6 (5.1–93.2)	<0.001	N = 56, 20.9 (1.7–88.6)	0.003
Serum cholesterol, mg/dL; median (range)	N = 241, 158 (64–304)	N = 201, 148 (64–288)	N = 40, 162 (94–304)	0.028	N = 77, 163 (66–272)	NS	N = 124, 144 (64–288)	NS
Abnormal cytogenetics, n. of patients (%)	N = 148, 46 (31)	N = 110, 39 (35.4)	N = 36, 5 (13.8)	0.025	N = 53, 12 (22.6)	0.008	N = 57, 27 (47.3)	0.008

§ P value refers to the difference between prefibrotic myelofibrosis and myelofibrosis-fibrotic type;

§§ P value refers to the difference between prefibrotic myelofibrosis and myelofibrosis-fibrotic type with bone marrow (BM) fibrosis grade 1;

§§§ P value refers to the difference between myelofibrosis-fibrotic type with BM fibrosis grade 1 and with BM fibrosis grade 2 or 3; ULN = Upper limit of normal; NS = Not significant.

### Determinants of Phenotypic Heterogeneity of Prefibrotic Myelofibrosis

Hematological characteristics varied among patients with pre-MF. Six out 79 (7.6%) female patients had hemoglobin greater than 16.5 g/dL, the value WHO system uses for classifying PV patients [11]. These patients received an original diagnosis of PV, but polyglobulia spontaneously remitted early and the BM picture was that of pre-MF. Sixty-nine percent of patients had more than 450×10^9^/L platelets, the value the WHO system uses for classifying ET patients [11]. One patient had thrombocytopenia (platelet count less than 150×10^9^/L), but the patient at the same time suffered by portal vein thrombosis that could be the cause of low platelet count. Three patients had white blood cell count equal or greater than 25×10^9^/L, and 2 patients had white blood cell count equal or lower than 4×10^9^/L.

By exploring the determinants of phenotypic heterogeneity at diagnosis, we documented that female sex was associated with younger age (mean, 35.8 yrs vs. 41.9 yrs; P = 0.003) and lower hemoglobin (mean, 13.9 g/dL vs. 14.5 g/dL; P = 0.028). *JAK2*V617F mutation (either heterozygous or homozygous) was associated with higher hemoglobin (mean, 14.4 vs. 13.5 g/dL; P = 0.004), lower platelet count (mean, 576 vs. 815×10^9^/L; P<0.001), higher spleen index (mean, 124.8 vs. 103.9 cm^2^; P = 0.006). Moreover, patients who carried a homozygous mutation had significantly higher white-blood cell count than those who either were wild-type or carried heterozygous mutation (mean, 13.3 vs. 8.41×10^9^/L; P<0.001). Splanchnic vein thrombosis at diagnosis or in the year preceding the diagnosis was associated with lower platelet count (mean, 374 vs. 716×10^9^/L; P<0.001), larger spleen index (mean, 140 vs.114 cm^2^; P = 0.004), and higher frequency of either heterozygous or homozygous *JAK2*V617F mutation (86.2% vs. 59.5%; P = 0.009).

Stepwise multiple regression analysis showed that *JAK2*V617F mutational status and male sex were both significant determinants of higher hemoglobin value (P = 0.016 and 0.042, respectively). Splanchnic vein thrombosis at diagnosis or in the year preceding diagnosis was the only determinant of lower platelet count (P = 0.029). Finally, having splanchnic vein thrombosis at diagnosis or in the year preceding the diagnosis and bearing *JAK2*V617F mutation were both significant determinants of higher spleen volume (P<0.001 and P = 0.024, respectively).

### Testing the Continuum Hypothesis

In order to test whether there was an underlying structure of PMF patients' characteristics that allowed to identify a continuum along which pre-MF was aligned, we categorized PMF-fibrotic type into two groups: PMF with early BM fibrosis (grade 1) and with advanced BM fibrosis (grade 2 or 3). Table 1 reports the clinical characteristics of these subgroups. A continuous modification was observed for male to female ratio, age, hemoglobin, platelet count and spleen size. However, only female to male ratio significantly steadily decreased (P = 0.03), while age steadily increased (P = 0.01) from pre-MF to PMF-fibrotic type with early and advanced myelofibrosis (ANOVA for trend). On the contrary, WBC did not differ among the three categories of patients and incidence of thrombotic events was mostly associated with the category of pre-MF.

CD34-positive cells in peripheral blood frequency and LDH concentration increased along the continuum of BM fibrosis, while CXCR4 expression and cholesterol level decreased (Table 3). Only the values of LDH and CXCR4 expression showed a significant trend (P = 0.033 and P = 0.003, respectively). Neither the frequency of *JAK2*V617F mutation nor the mutation allele burden changed among the three categories of patients.

Chromosomal aberrations steadily increased in frequency from pre-MF to early to advanced BM-fibrosis subtypes of PMF-fibrotic type. However, the trend was not statistically significant.

### Course of the Disease

**Figure 1:**
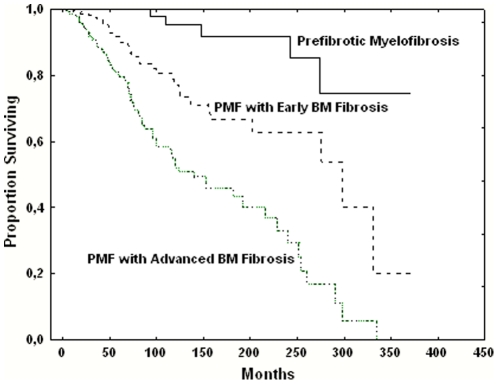
Survival of patients with primary myelofibrosis. Patients were categorized according they were diagnosed with prefibrotic myelofibrosis, myelofibrosis-fibrotic type with early bone marrow fibrosis (grade 1) or myelofibrosis-fibrotic type with advanced bone marrow fibrosis (grade 2 or 3).

The whole cohort of patients with PMF was followed for a median of 43 months (range, 1 to 375 months) after the diagnosis, representing 3848 person-years. The median survival of the entire cohort was 21 years. The cohort of patients with pre-MF was followed for a median of 66 months (range, 1 to 371 months) after the diagnosis. During 972 cumulative person-years of follow-up, 3.8% of patients with pre-MF died, while the proportion of patients with PMF-fibrotic type who died was 20.8%. The cause of death was blast transformation in 4 out 5 (80%) and 52 out 114 (45.6%), respectively. The median survival was not reached in pre-MF, while it was 16.6 years in PMF-fibrotic type (P<0.001). Ninety-eight, 81 and 56 percent of patients with pre-MF, PMF-fibrotic type with early and PMF-fibrotic-type with advanced BM fibrosis, respectively, were alive at 10 years from the diagnosis (P<0.001)(Figure 1).

The progression of BM fibrosis was studied in 67 (50.7%) patients with pre-MF. The 50% cumulative probability of progression from fibrosis grade 0 to fibrosis grade 1 was reached at 9.6 years and to fibrosis grade 2 or 3 at 16.4 years.

## Discussion

By investigating a large data-base of consecutive patients with PMF collected during a period of 21 years, we identified 132 cases with pre-MF. In order to make our population of pre-MF patients more homogenous and their distinction from PMF-fibrotic type clearer, we relied on the diagnostic clear-cut feature of absence of any degree of BM fibrosis. In this way, the possibility that the claimed variability in the reading of BM samples could have influenced our results still exists, but it should be minimal.

This study revealed that pre-MF possesses several epidemiological and clinical features not previously come to light. In comparison with patients with PMF-fibrotic type, the patients with pre-MF were more likely to be female, to be under the age of 50 at diagnosis, to have a milder hematological and clinical phenotype. Moreover, with the diagnosis of pre-MF we selected a category of patients with very high frequency (30%) of coincidental thrombotic events, mainly in splanchnic area. These results underscore the importance of the effects of age and sex on the phenotype of PMF, adding value to similar observations reported in patients with PV and ET [27], [28].

Despite the narrow criteria used for the diagnosis, our cases of pre-MF displayed different hematological presentations. Seventy percent of cases presented with an isolated thrombocytosis simulating ET, and 30% were diagnosed in coincidence with splanchnic vein thrombosis associated without any peripheral sign of myeloproliferation. This latter presentation coincides with that of masked MPNs associated with splanchnic vein thrombosis [29]–[33], and in particular of the variant “atypical myeloproliferative disorder with high thrombotic risk and slow disease progression" we first described in 1996 [34]. We documented that sex, *JAK2*V617F mutation and presence of splanchnic vein thrombosis were the most influential factors for the varying of phenotypes.

It resulted that the frequency of CD34-positive cells in peripheral blood, CXCR4 expression on CD34-positive cells, serum cholesterol and LDH levels were significantly different in pre-MF as compared with PMF-fibrotic type. Cytogenetic analysis confirmed that pre-MF is a less genetically unstable disease by showing that pre-MF had significantly lower frequency of chromosomal abnormalities as compared with PMF-fibrotic type.

We questioned whether it is justified to include pre-MF in the realm of PMF, as claimed by the WHO classification system. We documented that PMF-fibrotic type patients with an early or advanced BM fibrosis were aligned with pre-MF to form three categories of patients whose major epidemiological, clinical, and biological characteristics steadily varied in a continuum. Moreover, the survival of the patients steadily decreased. We further supported the contention that pre-MF is a prodromic phase of PMF by showing in pre-MF patients a progressive accumulation of reticulin fibers in the BM, thus documenting that transformation in PMF-fibrotic type is part of the natural history of the disease.

The evidence that pre-MF is a prodromic phase of PMF makes us more aware about the biology of the disease. Most of our cases of pre-MF had increase in BM myeloproliferation with megakaryocyte hyperplasia and dysplasia as the sole biological deviation from normal and many of them had thrombocytosis as the sole hematologic alteration. This addresses to the idea that the initial derangement of PMF could be restricted to megakaryopoiesis, and that dysmegakaryopoiesis may occur without any bone marrow fibrosis or hematopoietic progenitor cells mobilization, previously considered specific biological markers of PMF.

The mechanism leading to high risk of splanchnic vein thrombosis in the population of pre-MF is unknown. A result of this study that could have pathogenetic relevance is the strict association between JAK2V617F mutation and splanchnic vein thrombosis. In fact, 82% of patients with splanchnic vein thrombosis bore JAK2V617F mutation with respect to 59.5% of patients without splanchnic vein thrombosis. This result addresses to hypothesize that the cooperation of young age, female sex, and *JAK2*V617F mutation could facilitate the development of a thrombophilic environment in the splanchnic veins.

The evidence that pre-MF conforms to a continuum model has a major implication for our knowledge on the overall clinical characteristics of PMF. PMF is commonly described as the most severe among MPNs, with median survival ranging from 4 to 10.6 years in modern series that did not include pre-MF [35]–[46]. In our series, the median survival is 21 years, extremely longer than previously reported. This is due primarily to pre-MF patients which account for approximately 20% of the cases of PMF and who bear a very good prognosis. However, our series contains a large proportion of cases (30%) with PMF-fibrotic type with an early BM fibrosis that contributes to prolong the mean survival of the population. This drives to question on the real epidemiology of the disease, and on how much selection and reporting biases could influence the results of published series.

In conclusion, we have provided evidence that pre-MF is a mode of presentation of PMF with an indolent phenotype and a very long survival. Efforts to understand the natural history and to improve prognosis prediction for PMF have in the past focused on the population of patients with fibrotic type. Patients with pre-MF clearly deserve similar attention.
